# Limited Sampling Strategy for Estimation of Mycophenolic Acid Exposure in Adult Chinese Heart Transplant Recipients

**DOI:** 10.3389/fphar.2021.652333

**Published:** 2021-04-12

**Authors:** Hongping Xiang, Hong Zhou, Jing Zhang, Yongfeng Sun, Yirong Wang, Yong Han, Jie Cai

**Affiliations:** ^1^ Department of Pharmacy, Union Hospital, Tongji Medical College, Huazhong University of Science and Technology, Wuhan, China; ^2^ Hubei Province Clinical Research Center for Precision Medicine for Critical Illness, Wuhan, China; ^3^ Department of Cardiovascular Surgery, Union Hospital, Tongji Medical College, Huazhong University of Science and Technology, Wuhan, China; ^4^ Department of Pharmacy, The Third People’s Hospital of Chengdu, Chengdu, China

**Keywords:** mycophenolic acid, pharmacokinetics, therapeutic drug monitoring, limited sampling strategy, heart transplantation

## Abstract

**Background:** With the increasing use of mycophenolic acid (MPA) formulations in organ transplantation, the need for personalized immunosuppressive therapy has become well recognized based on therapeutic drug monitoring (TDM) for avoidance of drug-related toxicity while maintaining efficacy. Few studies have assessed area under the 12 h concentration-time curve of MPA (MPA-AUC_0–12h_) in heart transplant recipients who received mycophenolate mofetil (MMF) dispersible tablets (MMFdt). The aim of the study was to investigate the pharmacokinetics (PK) of MMFdt combined with tacrolimus and further to develop a practical method for estimation of MPA-AUC_0–12h_ using a limited sampling strategy (LSS).

**Methods:** A prospective study in a single center was performed in patients who continuously administrated with MMFdt or MMF capsule (MMFc) for at least 7 days after cardiac transplantation from 2018 to 2020. A total of 48 Chinese adult heart transplant recipients were enrolled. Blood samples were collected before and 0.5, 1, 1.5, 2, 4, 6, 8, 10 and 12 h after MMF administration. The validated high-performance liquid chromatography combined with tandem mass spectrometry method was used to measure MPA concentrations. Non-compartmental pharmacokinetic (PK) analysis was applied to calculate the data obtained from individual recipients by WinNonlin. LSS models were developed for MPA-AUC_0–12h_ prediction with multivariate stepwise regression analysis.

**Results:** A large inter-individual variability was observed in AUC_0–12h_, T_max_, C_max_, MRT_0–12h_, t_1/2_ and CL/F after multiple dosing of MMFdt. However, no significant differences were observed between main PK parameters of MMFdt and MMFc. The best estimation of MPA-AUC_0–12h_ was achieved with four points: MPA-AUC_0–12h_ = 8.424 + 0.781 × C_0.5_ + 1.263 × C_2_ + 1.660 × C_4_ + 3.022 × C_6_ (*R* ^2^ = 0.844). The mean prediction error (MPE) and mean absolute prediction error (MAPE) of MPA-AUC_0–12h_ were 2.09 ± 14.05% and 11.17 ± 8.52%, respectively. Both internal and external validations showed good applicability for four-point LSS equation.

**Conclusion:** The results provide strong evidence for the use of LSS model other than a single time-point concentration of MPA when performing TDM. A four-point LSS equation using the concentrations at 0.5, 2, 4, 6 h is recommended to estimate MPA-AUC_0–12h_ during early period after transplantation in Chinese adult heart transplant recipients receiving MMFdt or MMFc. However, proper internal and external validations with more patients should be conducted in the future.

## Introduction

Mycophenolic acid (MPA) is commonly used in combination with calcineurin inhibitors (CNIs), including tacrolimus (TAC) or cyclosporin A (CsA), and glucocorticoids to form a triple immunosuppressive treatment regimen for the prophylaxis of organ rejection. MPA exerts the pharmacological effects by selectively and reversibly inhibiting inosine monophosphate dehydrogenase (IMPDH), thereby preventing denovo synthesis of purine and finally the proliferation of both T and B lymphocytes (Lipsky, 1996; Allison and Eugui, 2000; Holt, 2002; Staatz and Tett, 2007). Currently, two mycophenolate compounds are available, mycophenolate mofetil (MMF) and enteric-coated mycophenolate sodium (EC-MPS). MMF is hydrolyzed in the stomach and by tissue and plasma esterases to release MPA which is absorbed in the stomach and proximal small intestine (Staatz and Tett, 2007). EC-MPS releases mycophenolate sodium in the gastrointestinal (GI) tract with a pH > 5.5, the sodium salt rapidly dissolving to MPA which is absorbed in the small intestine (Nowak and Shaw, 1995). In China, three oral formulations of MMF are commonly used, including tablets, capsules and dispersible tablets, while MMF was initially marketed as a “one-dose-suits-all” drug with a fixed dose of 1 ∼ 2 g daily for organ transplantation patients regardless of the formulations (Pharma, 2014). Results from clinical trials demonstrated that MMF administered according to a fixed-dosing regimen without monitoring can improve the graft survival in patients with renal, heart, or liver transplantation (Ojo et al., 2000; Hosenpud and Bennett, 2001; Lake et al., 2005).

However, MPA shows a large inter-individual variability in pharmacokinetic (PK), resulting in an over 10-fold range in MPA exposure (expressed as area under the total MPA concentration-time curve from 0 to 12 h [AUC_0–12h_]) (MPA-AUC_0–12h_) (Weber et al., 1999; Shaw et al., 2000a; Ensom et al., 2003; Staatz and Tett, 2007). MPA has a narrow therapeutic window that patients would display significant associations between high concentrations with serious adverse drug reactions and low concentrations with ineffectiveness. Several studies revealed that MPA exhibits nonlinear pharmacokinetics, with bioavailability decreasing significantly with increasing doses, perhaps due to saturable absorption processes or saturable enterohepatic recirculation (Johnson et al., 1999; Shaw et al., 2000b; Squifflet et al., 2001; Armstrong et al., 2005; Zhao. et al., 2019). Additionally, serum albumin levels, liver and renal functions, genetic polymorphisms as well as drug–drug interactions were some vital factors that are able to hugely influence the PK of MPA (Staatz and Tett, 2007; Guo et al., 2018). In terms of these findings, MMF is more suitable for individualized dosing regimens based on therapeutic drug monitoring (TDM) rather than fixed dose (Kuypers et al., 2010). The MPA-AUC_0–12h_ has been recommended for dose adjustment in clinical application with a better correlation with the efficacy of MPA when compared with the single-point concentration of MPA (Baraldo et al., 2005; Dösch et al., 2006; Chaabane et al., 2013; Cai et al., 2015). A defined target range of MPA-AUC_0–12h_ within 30–60 μg·h/mL detected by high performance liquid chromatography (HPLC) was commonly used for kidney transplantation recipients (Bennett, 2003). Measurements of a single concentration or AUC values have been suggested in cardiac transplantation (DeNofrio et al., 2000; Mardigyan et al., 2005). An exposure-effect association study demonstrated that an AUC threshold of 50 μg·h/mL was proposed for adult heart transplant recipients (Woillard et al., 2015). Routine monitoring of MPA-AUC_0–12h_ requires intensive blood sampling, which is hardly available in clinical practice due to the prolonged time, poor compliance or high costs. Ratain et al. and Egorin et al. were the earliest to present that PK parameter measurements could be simplified by using a technique called limited sampling strategy (LSS) (Ratain and Vogelzang, 1987; Ratain et al., 1988; Egorin et al., 1989). The LSS is recommend as a potential method to predict the MPA-AUC_0–12h_ with 2–4 concentrations by using multiple regression analysis (MRA) or the maximum a posteriori Bayesian method for model development (Staatz and Tett, 2007).

Currently, LSS models of MPA have been widely reported in patients with liver and kidney transplantation (Ting et al., 2006; Hao et al., 2007; Jiao et al., 2007; Zhou et al., 2007; Zicheng et al., 2007; Bruchet and Ensom, 2009; Gu et al., 2012). However, data on heart transplantation is still rare. There have been some researches of LSS models of MPA combined with CsA in heart transplant patients (Monchaud et al., 2003; Baraldo et al., 2005; Dösch et al., 2006; Pawinski et al., 2009). CsA inhibits biliary excretion of 7-O-MPA-glucuronide (MPAG) by multidrug resistance-associated protein 2 (MRP-2), reducing enterohepatic recirculation of MPA (van Gelder et al., 2001; Deters et al., 2005; Saitoh et al., 2006). Exposure to MPA when MMF is given in combination with CsA is approximately 30–40% lower than when given alone or with TAC or sirolimus, which indicate that the LSS models of MPA would vary with the combination of different drugs (Filler et al., 2000; Staatz and Tett, 2007). At present, only two LSS studies for estimation of MPA-AUC_0–12h_ in heart transplant recipients with MMFc and TAC therapy were reported with relatively small populations (Wada et al., 2007; Kaczmarek et al., 2008). Therefore, a new model to determine exposure of MPA combined with TAC in Chinese heart transplant recipient population assessed by LSS method is necessary in order to facilitate individualized therapy.

Previously, the bioequivalence and PK characteristics of MMF dispersible tablets (MMFdt) and capsules (MMFc) had no hugely differences in healthy Chinese male volunteers (Zhang et al., 2010). Little information was obtained about PK profile and LSS models of MMFdt in heart transplant patients. The aim of the present study was to investigate the PKs of MMF formulations combined with TAC in adult patients after heart transplantation, and further to establish a LSS model for MPA-AUC_0–12h_ estimation.

## Materials and Methods

### Patients

Adult heart transplant patients (aged 18–70 years) from the Department of Cardiovascular Surgery, Union Hospital of Tongji Medical College, Huazhong University of Science and Technology, who met the following inclusion criteria, were enrolled in the present study. The inclusion criteria were as follows: heart transplantation for the first time, triple immunosuppressive treatment consisting of MMFdt (Huadong Pharmaceutical Group Co., Ltd., Hangzhou, Zhejiang, China) or MMFc (Shanghai Roche Pharmaceuticals Ltd., Shanghai, China), TAC (Astellas Ireland Co., Ltd., Tokyo, Japan) and prednisone (Wuhan Yuanda Pharmaceutical Group Co., Ltd., Wuhan, Hubei, China), treatment with 750 mg MMFdt or MMFc every twelve hours for more than 7 days. Exclusion criteria were: second heart transplantation, co-medication with an immunosuppressive agent other than TAC, MMFdt or MMFc and prednisone, suspected noncompliance, severe adverse drug reactions related to immunosuppressive drugs, especially gastrointestinal reactions, confirmed acute rejection or infection. The study was approved by the Ethics Committee of the Union Hospital of Tongji Medical College, Huazhong University of Science and Technology and all patients signed their written, informed consents.

### Study Design

The patients were continuously administrated with MMFdt or MMFc for at least 7 days after cardiac transplantation. Each patient was given MMFdt or MMFc at the dose of 750 mg every 12 h. During the PK study, all patients received liquid soft food at the prescribed time. Blood samples were taken before (0 h) and 0.5, 1, 1.5, 2, 4, 6, 8, 10 and 12 h after administration. The blood samples were collected in ethylenediaminetetraacetic acid tubes and immediately separated the plasma after centrifuging at 2,333 g for 8 min. All the samples were stored at −80°C for MPA determination by a high-performance liquid chromatography combined with tandem mass spectrometry (LC-MS/MS) method, which was validated according to the verification guidelines for quantitative analysis methods of biological samples in Chinese Pharmacopoeia (version 2015).

### Mycophenolic Acid Concentration Determination

MPA concentrations were measured using the validated LC-MS/MS method which was established previously (Zhao. et al., 2019). In brief, an Agilent ZORBAX SB-C18 (3.5 μm, 2.1 × 50 mm) column was used for separation and MPA-d_3_ was used as the internal standard. The mobile phase was 0.005 mol/L ammonium acetate (0.25% formic acid) aqueous solution and acetonitrile, working in a gradient manner with a flow rate of 0.2 ml/min. Multiple reactions monitoring (MRM) mode was adopted and electrospray ionization was a negative-ion. The precursor ion → product ion transitions m/z was 319.1→191.0 for MPA and m/z 322.1→191.1 for MPA-d_3_, respectively. The standard curve range for MPA determination is 0.1–40.0 μg/mL. The lower limit of quantitation was 0.1 μg/mL. The intra- and inter-day imprecision was less than 10%; with the intra-day precision d from 4.0 to 7.3% and the inter-day precision from 2.5 to 6.2%.

### Pharmacokinetic Analysis

Non-compartmental PK parameters were derived from each individual plasma MPA concentration–time profile using WinNonlin Professional (v.5.2) software (Pharsight Corporation, Mountain View, CA, United States). The corresponding AUC_0–12h_ were calculated by the linear-trapezoidal rule. C_max_ was defined as the maximum daytime MPA concentration after dosing with MPA within the dosing interval. T_max_ was defined as the time to reach the maximum daytime MPA concentration. MRT was defined as mean residence time. t_1/2_ was defined as the time required for the highest concentration of the drug in plasma to decrease by half. CL/F was defined as the plasma volume of the drug removed per hour.

### Development and Validation of Limited Sampling Strategy Models

MPA-AUC_0–12h_ LSS models were established using MRA by the following procedure. Step 1: Random number table was used to divide patients received MMFdt into model building and validation groups. Step 2: The value of measured MPA-AUC_0–12h_ (MPA-AUC_0-12h-observed_) was regarded as a dependent variable and the concentration of MPA at each time point was used as independent variable. MPA concentration at each sampling time was correlated by linear regression analysis with the MPA-AUC_0–12h_ in model building group. Step 3: Multiple stepwise linear regression analysis was performed to give improved correlations with total MPA-AUC_0–12h-observed_. These models can be expressed as: MPA-AUC_0–12h_ = X (intercept, constant value) + *b*_1_ × *c*_1_ + ⋯ + b_n_ × c_n,_ where b_1_… , b_n_ are regression coefficients, n is the nominal sample collection time, and c_1_ … , c_n_ are concentration values measured at times 1 through n. The correlation coefficient (*R* ^2^) was used to evaluate the regression level of the equation. The prediction bias of these LSS-derived equations was quantified as the percentage of mean prediction error (MPE) and mean absolute prediction error (MAPE). MPA-AUC_0–12h-predicted_ was defined as the value of predicted MPA-AUC_0–12h_. The two error parameters were calculated by the following equations:

$$\mathrm{MPE}\%=\frac{1}{n}\sum_{i=1}^{n}\left\{\left(\mathrm{AU}C_{0-12h-\mathrm{predicted}}-\mathrm{AU}C_{0-12h-\mathrm{observed}}\right)/\mathrm{AU}C_{0-12h-\mathrm{observed}}\right\}\times 100$$

$$\mathrm{MAPE}\%=\frac{1}{n}\sum_{i=1}^{n}\left\{\left|\left(\mathrm{AU}C_{0-12h-\mathrm{predicted}}-\mathrm{AU}C_{0-12h-\mathrm{observed}}\right)/\mathrm{AU}C_{0-12h-\mathrm{observed}}\right|\right\}\times 100$$

Step 4: The nonparametric regression proposed by Passing-Bablok regression was used to estimate the relationship between MPA-AUC_0–12h-predicted_ and MPA-AUC_0–12h-observed._ The Bland–Altman difference plots were adequate for estimating bias between MPA-AUC_0-12h-predicted_ and MPA-AUC_0-12h-observed_. The best model was selected based on account the values of *R* ^2^, predictive bias, and the Bland-Altman analysis.

Model estimation was conducted by both internal and external validation after obtaining the candidate equations. Bootstrap method was used for internal validation. Another set of data is used for external validation.

### Statistical Analysis

All data were statistically analyzed using IBM SPSS Statistics Version 22.0 (SPSS Inc., Chicago, IL, United States) and GraphPad Prism software v8.0.2 (GraphPad Software, San Diego, California, United States). Results are expressed as the mean ± standard deviation, or median (95% confidence interval). Kruskal Wallis test was used for nonparametric test of three or four independent samples. *p* < 0.05 was considered statistically significant. The Passing-Bablok regression and Bland-Altman analysis were used to evaluate the consistency between MPA-AUC_0–12h-predicted_ and MPA-AUC_0–12h-observed_.

## Results

### Patient Demographics

A total of 48 Chinese adult heart transplant recipients (39 males, 9 female) were enrolled between January 1, 2018 and January 1, 2020, among them, 42 patients received MMFdt and the rest population received MMFc. The main characteristics of the study population based on model development and model validation, patients receiving MMFc for model validation (MMFc group) were listed in Table 1.

**TABLE 1:** The demographic and clinical data of patients in model development and validation groups.

Variates	Model development (*n* = 30)	Model validation (*n* = 12)	Total (*n* = 42)	MMFc (*n* = 6)	*p*
Sex (male/female) (case)	25/5	10/2	35/7	4/2	0.79
Age (years)	47.20 ± 11.82	49.50 ± 10.56	47.86 ± 11.39	52.83 ± 4.79	0.73
Body weight (kg)	64.77 ± 12.29	65.41 ± 11.80	64.96 ± 11.99	60.40 ± 10.78	0.88
WBC (10^9^/L)	10.24 ± 2.77	10.48 ± 3.73	10.31 ± 3.03	11.37 ± 5.38	1.00
Neutrophils (10^9^/L)	9.01 ± 2.93	9.01 ± 3.71	9.01 ± 3.13	8.88 ± 3.24	0.96
Hb (g/L)	95.42 ± 22.95	105.92 ± 16.56	98.42 ± 21.66	98.83 ± 15.54	0.83
HCT (%)	28.59 ± 5.82	31.88 ± 4.99	29.53 ± 5.74	28.53 ± 4.45	0.63
ALT (U/L)	34.61 ± 24.66	62.73 ± 62.83	42.65 ± 40.68	31.83 ± 36.26	0.27
AST (U/L)	39.78 ± 45.65	26.42 ± 17.79	35.96 ± 39.96	17.83 ± 12.32	0.43
ALB (g/L)	36.73 ± 14.58	29.64 ± 11.75	34.70 ± 14.07	37.90 ± 4.75	0.83
BUN (mmol/L)	13.87 ± 11.13	14.76 ± 10.96	14.13 ± 10.96	11.38 ± 2.59	0.88
Cr (umol/L)	93.33 ± 80.83	54.73 ± 25.02	82.30 ± 71.42	95.58 ± 58.29	0.18
CCr (ml/min)	112.63 ± 61.25	148.84 ± 58.06	123.40 ± 61.83	77.14 ± 35.86	0.16
Dose of TAC (mg/q12 h)	2.24 ± 0.58	2.01 ± 0.71	2.18 ± 0.62	2.25 ± 0.45	0.70
Dose of PDN (mg/q12 h)	24.83 ± 7.65	24.79 ± 5.69	24.82 ± 7.08	21.25 ± 5.42	0.50
Dose of MPA (mg/q12 h)	750	750	750	750	1.00
Trough concentration of TAC (μg/L)	7.12 ± 2.95	8.74 ± 4.03	7.59 ± 3.32	11.73 ± 3.87	0.08
Sampling time after MPA initiation (d)	8.57 ± 1.68	8.29 ± 1.23	8.49 ± 1.56	8.67 ± 1.60	1.00

MMFc, mycophenolate mofetil capsule; WBC, white blood cell; Hb, hemoglobin; HCT, hematocrit; ALT, alanine aminotransferase; AST, aspartate aminotransferase; ALB, albumin; BUN, blood urea nitrogen; Cr, creatinine; CCr, creatinine clearance; TAC, tacrolimus; PDN, prednisolone; MPA, mycophenolic acid.

### Pharmacokinetic Characteristics of MMFdt and MMFc

**FIGURE1:**
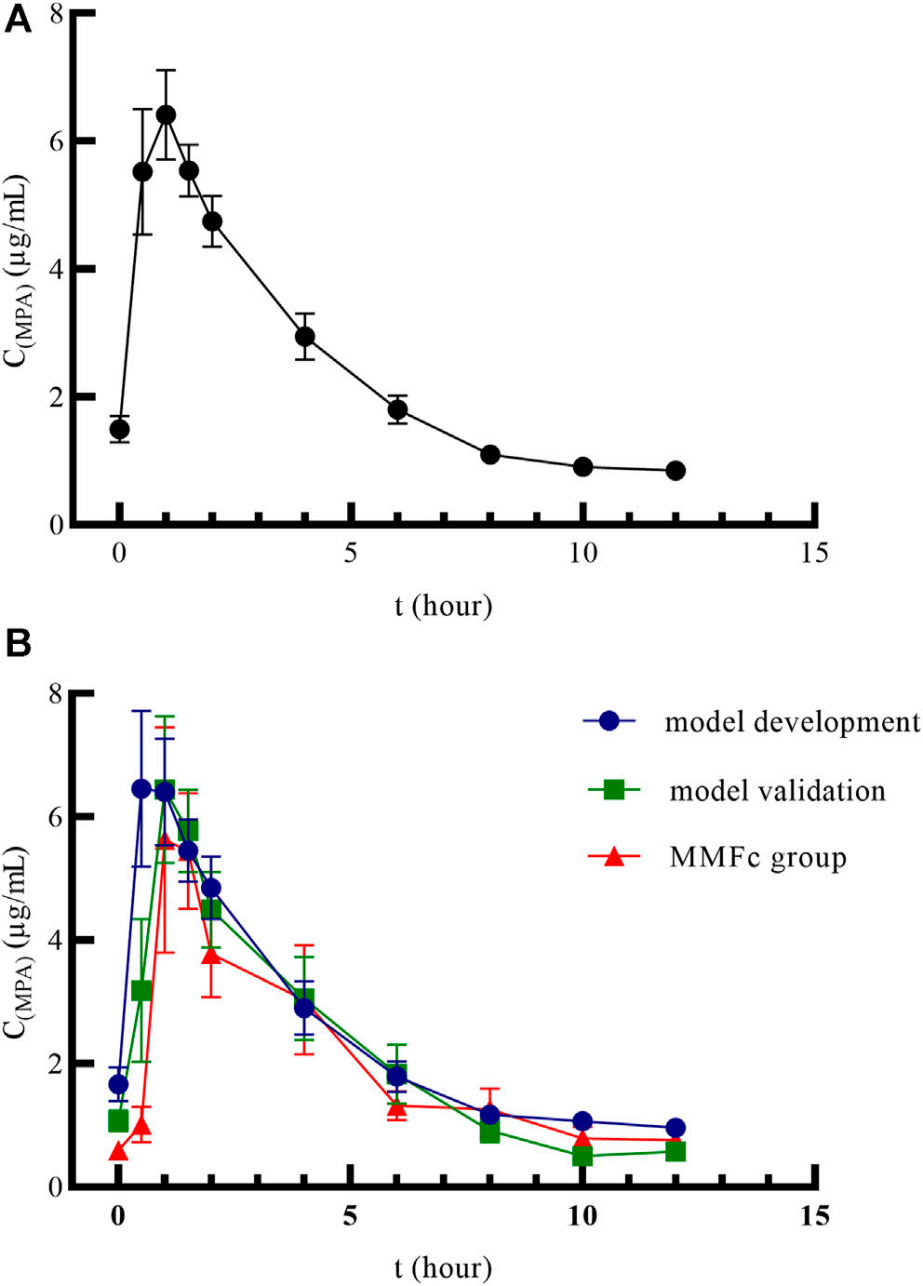
Mean plasma concentration–time curves of MMFdt in all patients **(A)**, model building group, model validation group and MMFc group **(B)** in heart transplant recipients. MMFdt, mycophenolic mofetil dispersible tablets; MMFc, mycophenolate mofetil capsule; MPA, mycophenolic acid; C_(MPA)_, concentration of MPA; t, time.

A total of 48 patients were included in the PK study. The mean plasma concentration–time curves of MPA after administration of MMFdt in all patients was shown in Figure 1A. The mean plasma concentration-time curves after administration of MMFdt or MMFc, including three groups: model building group, model validation group and MMFc group, were shown in Figure 1B. These three-plasma concentration-time curves were single peaks that appeared at 0.5 ∼ 2 h after oral administration. C_max_ in model building group (10.09 ± 6.44 μg/mL) and model validation group (8.49 ± 3.47 μg/mL) of MMFdt were slightly higher than that of MMFc group (7.48 ± 3.22 μg/mL). Additionally, second peaks were observed in some patients between 5 ∼ 12 h after taking MMFdt (Supplementary Figure S1A) or MMFc (Supplementary Figure S1B).

An overview of the PK parameters of model building group, model validation group, all MMFdt patients and MMFc group were shown in Table 2. The main PK parameters including AUC_0–12h_, T_max_, C_max_, MRT_0–12h_, t_1/2_ and CL/F were all similar between those four groups. The inter-individual variability of all MMFdt in AUC_0–12h_, T_max_, C_max_, MRT_0–12h_, t_1/2_ and CL/F were 31.45, 71.84, 59.01, 21.60, 68.38, 38.60% respectively. Among all MMFdt patients, 25 recipients (59.5%) had an MPA-AUC_0–12h_ value lower than 30 μg·h/mL, and other patients were within 30 ∼ 60 μg·h/mL. In MMFc group, most of the recipients (*n* = 5, 83.3%) had an MPA-AUC_0–12h_ below 30 μg·h/mL and only one recipient achieved the targeted range (30 ∼ 60 μg·h/mL). No patient had an MPA-AUC_0–12h_ value >60 μg·h/mL both in MMFdt and MMFc groups.

**TABLE 2:** Pharmacokinetic parameters of MMFdt and MMFc in heart transplant recipients.

Parameter	Model development (*n* = 30)	Model validation (*n* = 12)	Total (*n* = 42)	MMFc (*n* = 6)	*p*
AUC_0–12h_ (μg·h/mL)	30.46 ± 10.09	26.80 ± 6.91	29.41 ± 9.36	25.03 ± 8.46	0.43
T_max_ (h)	1.85 ± 1.41	1.75 ± 1.14	1.82 ± 1.32	2.17 ± 1.44	0.96
C_max_ (µg/mL)	10.09 ± 6.44	8.49 ± 3.47	9.63 ± 5.75	7.48 ± 3.22	0.81
MRT_0–12_ (h)	3.92 ± 0.90	3.58 ± 0.63	3.82 ± 0.83	4.04 ± 1.00	0.59
t_1/2_ (h)	7.59 ± 5.68	6.78 ± 3.31	7.36 ± 5.09	11.16 ± 12.45	0.99
CL/F (L/h)	20.78 ± 8.63	24.92 ± 8.03	21.96 ± 8.58	25.13 ± 13.56	0.29

MMFdt, mycophenolic mofetil dispersible tablets; MMFc, mycophenolate mofetil capsule; AUC_0–12h_, area under the curve from 0 to 12 h; T_max_, time to reach maximum peak plasma concentration, C_max_, maximum plasma drug concentration; MRT_0–12,_ mean residence time; t_1/2_, half-life; CL/F, apparent total clearance of the drug from plasma after oral administration.

### Limited Sampling Strategy Models of MPA-AUC_0–12h_

The results of regression equations obtained by sampling time points and MPA-AUC_0–12h_ were shown in Table 3. The equation showed that only C_6_ was in a week correlation (*R* ^2^ = 0.428) with MPA-AUC_0–12h_.

**TABLE 3:** Univariate correlation between the MPA plasma concentration at each time point and MPA-AUC_0–12 h_ (*n* = 30).

Time point (h)	Model equation	*R* ^2^	MPE (%)	MAPE (%)	Difference between LSS and full AUC values (case)		
					Within ±15%	Within ±20%	Within ±25%
0	Y = 4.124C_0_ + 22.964	0.385	8.58 ± 33.85 (−32.31, 97.91)	25.37 ± 23.59 (1.76, 97.9)	11	15	19
0.5	Y = 0.554C_0.5_ + 26.248	0.146	10.49 ± 36.11 (−45.75, 104.46)	27.78 ± 24.89 (0.89, 104.46)	11	16	19
1	Y = 0.530C_1_ + 26.430	0.063	11.53 ± 38.69 (−46.80, 109.54)	29.56 ± 27.03 (0.13, 109.54)	12	16	17
1.5	Y = 1.890C_1.5_ + 19.523	0.274	8.59 ± 31.02 (−41.10,76.57)	23.59 ± 21.52 (0.32, 76.57)	13	16	20
2	Y = 1.549C_2_ + 22.314	0.184	9.94 ± 35.16 (−44.28–90.03)	29.83 ± 25.20 (0.56, 90.03)	13	18	19
4	Y = 2.319C_4_ + 23.093	0.304	9.78 ± 37.52 (−33.97, 97.64)	27.17 ± 27.52 (1.39, 97.64)	13	15	20
6	Y = 4.807C_6_ + 21.214	0.428	7.96 ± 32.91 (33.32, 97.54)	24.63 ± 22.82 (0.63, 97.54)	11	13	20
8	Y = 7.528C_8_ + 21.020	0.230	10.03 ± 38.21 (−41.21, 111.76)	26.53 ± 28.91 (0.36, 111.76)	11	15	19
10	Y = 5.469C_10_ + 23.979	0.119	11.28 ± 40.13 (−44.26, 118.70)	28.79 ± 29.74 (1.08, 118.70)	12	15	19
12	Y = 6.238C_12_ + 23.820	0.129	11.45 ± 43.14 (−45.76, 164.95)	26.64 ± 35.53 (0.00, 164.95)	15	19	19

MPA, mycophenolic acid; MPA-AUC_0–12h,_ the area under the 12-h concentration-time curve of MPA; *R* ^2^, correlation coefficient; MPE, mean prediction error; MAPE, mean absolute prediction error.

**FIGURE 2:**
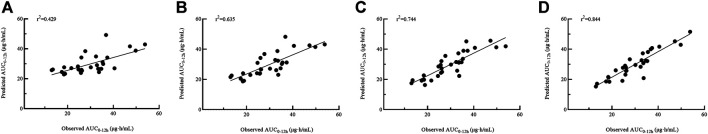
Passing–Bablok regression analysis between the observed AUC_0–12h_ and the predicted AUC_0–12h_ of MPA obtained from model building group using model 1 **(A)**, model 2 **(B)**, model 3 **(C)** and model 4 **(D)**. AUC_0–12h_, the area under the 12 h concentration-time curve; MPA, mycophenolic acid.

**FIGURE 3:**
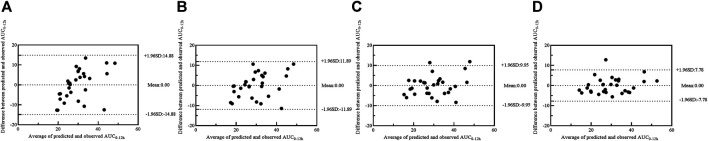
Bland–Altman plot of differences between the observed AUC_0–12h_ and the predicted AUC_0–12h_ of MPA in model building group using model 1 **(A)**, model 2 **(B)**, model 3 **(C)** and model 4 **(D)**. AUC_0–12h_, the area under the 12-h concentration-time curve; MPA, mycophenolic acid.

Four models with 1-point (model 1), 2-point (model 2), 3-point (model 3) and 4-point (model 4) LSS equations were later developed to estimate MPA-AUC_0–12h_. The model formula, *R* ^2^, prediction error of the above models and difference between LSS and full AUC values were shown in Table 4. The MPE and MAPE of model 4 were 2.09 ± 14.05% and 11.17 ± 8.52%, respectively. The abilities of the four models to predict the MPA-AUC_0–12h_ were depicted in Figure 2. The Bland-Altman analysis was presented in Figure 3. One case in model 4 (Figure 3D) exceed 95% confidence interval (CI) while model 1 (Figure 3A) and model 2 (Figure 3B) were both within the 95% CI and model 3 (Figure 3C) had 2 cases exceeding 95% CI. Based on the above analysis, the 4-point equation (C_0.5_, C_2_, C_4_ and C_6_) was the best LSS model: MPA-AUC_0–12h_ = 8.424 + 0.781 × C_0.5_ + 1.263 × C_2_ + 1.660 × C_4_ + 3.022 × C_6_ (*R* ^2^ = 0.844).

**TABLE 4:** LSS models of MPA-AUC_0–12 h_ using multiple linear regression analysis (n = 30).

Models	Time point (h)	Model equation	*R* ^2^	MPE (%)	MAPE (%)	Difference between LSS and full AUC values (case)		
						Within ±15%	Within ±20%	Within ±25%
1	6	Y = 4.807C_6_ + 21.214	0.428	7.96 ± 32.91 (−33.32, 97.54)	24.63 ± 22.82 (0.63, 97.54)	11	13	20
2	6, 0.5	Y = 5.165C_6_ + 0.663C_0.5_ + 16.294	0.635	4.88 ± 24.10 (−31.49, 67.19)	17.63 ± 16.84 (1.38, 67.19)	15	20	22
3	6, 0.5, 2	Y = 4.679C_6_ + 0.691C_0.5_ + 1.220C_2_ + 11.066	0.744	3.02 ± 17.84 (−33.76,44.34)	14.14 ± 10.99 (1.05,44.34)	18	21	26
4	6, 0.5, 2, 4	Y = 3.022C_6_ + 0.781C_0.5_ + 1.263C_2_ + 1.660C_4_ + 8.424	0.844	2.09 ± 14.05 (−38.09, 27.37)	11.17 ± 8.52 (0.45, 38.09)	22	25	28
5	6, 0.5, 2, 4, 1	Y = 3.312C_6_ + 0.615C_0.5_ + 1.059C_2_ + 2.040C_4_ + 0.765C_1_ + 3.966	0.942	0.55 ± 9.07 (−18.96, 23.96)	6.78 ± 5.92 (0.36, 23.96)	26	29	30

MPA, mycophenolic acid; MPA-AUC_0–12h,_ the area under the 12-h concentration-time curve of MPA; *R* ^2^, correlation coefficient; MPE, mean prediction error; MAPE, mean absolute prediction error.

### Model Validation

Internal validation was estimated with bootstrapping, where 1,000 sets of data were generated from the original data to evaluate the accuracy of each parameter of the best LSS model. The parameters of model 4 were all within the parameter estimates by repeated sampling, indicating that the accuracy and robustness were acceptable.

**FIGURE 4:**
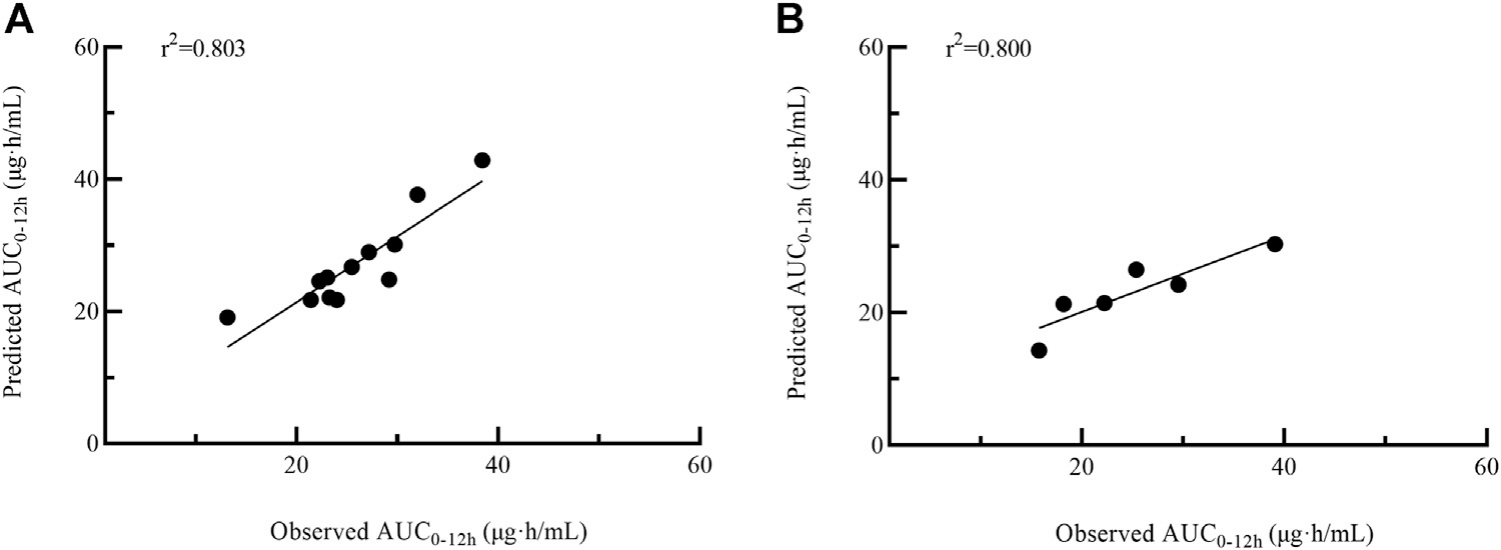
Passing–Bablok regression analysis between the observed AUC_0–12h_ and the predicted AUC_0–12h_ of MPA obtained from model validation group **(A)** and MMFc group **(B)** by model 4. AUC_0–12h_, the area under the 12 h concentration-time curve; MPA, mycophenolic acid; MMFc, mycophenolate mofetil capsule.

**FIGURE 5:**
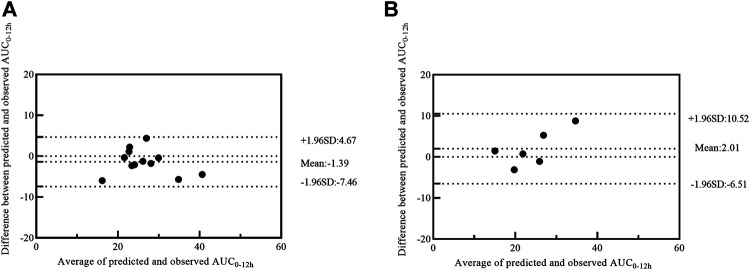
Bland–Altman plot of differences between the observed AUC_0–12h_ and the predicted AUC_0–12h_ of MPA in model validation group **(A)** and MMFc group **(B)** by model 4. AUC_0–12h_, the area under the 12 h concentration-time curve; MPA, mycophenolic acid; MMFc, mycophenolate mofetil capsule.

Twelve patients were used for further model external validation. The value of *R* ^2^ (Passing-Bablok regression) and prediction error were shown in Table 5. The MPE and MAPE of model validation group were 6.67 ± 15.36% and 11.52 ± 11.82%, respectively. The ability of the final model was shown in Figure 4A. Good agreement was confirmed between MPA-AUC_0–12h-predicted_ and MPA-AUC_0–12h-observed_ (Figure 5A), and none of values were outside the 95% CI.

**TABLE 5:** External validation of model 4 using model validation group and MMFc group.

Validation groups	*R* ^2^	MPE (%)	MAPE (%)	Difference between LSS and full AUC values (case)		
				Within ±15%	Within ±20%	Within ±25%
MMFdt group (*n* = 12)	0.803	6.67 ± 15.36 (15.01, 45.59)	11.52 ± 11.82 (1.38, 45.59)	10	11	11
MMFc group (*n* = 6)	0.800	−5.25 ± 14.62 (−22.44, 17.15)	12.41 ± 7.88 (3.37, 22.44)	3	5	6

MMFc, mycophenolate mofetil capsule; *R* ^2^, correlation coefficient by Passing-Bablok regression; MPE, mean prediction error; MAPE, mean absolute prediction error.

Given the similar in PK profile between MMFdt and MMFc in our population, MMFc group was also used to validate the feasibility of 4-point LSS equation in heart transplant patients. The MPE and MAPE in MMFc group were -5.25 ± 14.62% and 12.41 ± 7.88%, respectively (Table 5). The consistency between MPA-AUC_0–12h-predicted_ and MPA-AUC_0-12h–observed_ were good with all patients were within the 95% CI (Figure 5B). The result of Passing-Bablok regression was shown in Figure 4B.

## Discussion

As for the narrow therapeutic window and the large inter-individual variability of MPA, TDM is essential for individual dose adjustment of MPA after transplantation (Shaw et al., 2000a; Staatz and Tett, 2007; Ferreira et al., 2020). MPA-AUC_0–12h_ is better than a single time-point concentration to surrogate the MPA exposure (Wada et al., 2007; Kaczmarek et al., 2008; Cai et al., 2018). Owing to the requirement for dense sampling, traditionally full MPA-AUC_0–12h_ monitoring is laborious. Therefore, LSS is recommended as an optimal method to estimate MPA-AUC_0–12h_ (Bruchet and Ensom, 2009). Recently, LSS models estimating MPA-AUC_0–12h_ has been reported in liver and kidney transplantation, but it is rare in heart transplantation (Ting et al., 2006; Hao et al., 2007; Jiao et al., 2007; Zicheng et al., 2007; Bruchet and Ensom, 2009). The present study was the first to report the PK characteristics of MMFdt in early heart transplant recipients, and also demonstrated a best four-point LSS model to estimate MPA-AUC_0–12h_ for the first time.

In the present study, large inter-individual variabilities in PK parameters including AUC_0–12h_, T_max_, C_max_, MRT_0–12h_, t_1/2_ and CL/F were observed both in patients receiving MMFdt and MMFc, which was in agreement with previous reports in patients with renal, heart, or liver transplantation (Wada et al., 2007; Zicheng et al., 2007; Zhang et al., 2018). T_max_ was higher, while C_max_, t_1/2,_ and AUC were relatively lower in our MMFdt population, which is slightly different from those in healthy volunteers (Zhang et al., 2010). It could possibly be explained by the fact that dosages, pathophysiological factors and sampling time in our patient cohort differed from that in healthy volunteers. T_max,_ C_max_ and CL/F in our study were similar to those in renal transplant patients receiving MMFdt (Cai et al., 2018; Zhang et al., 2018). Enterohepatic recirculation (EHR) is an important characteristic of MPA PK profile, which will lead to the second peak of MPA concentration–time curves and accounted for 40–50% of AUC (Bullingham et al., 1996a; Staatz and Tett, 2007; Zhang et al., 2010). Usually, a second peak was identified in 6–12 h after MMF administrated orally (Bullingham et al., 1998). Uridine diphosphate glucuronosyltransferases (UGTs) metabolize MPA into MPAG which has no pharmacological activity with respect to inhibition of IMPDH via glucuronidation in the GI tract, liver and kidney (Bowalgaha and Miners, 2001; Picard et al., 2005). MPAG, the main metabolite, excreted into the bile may be deconjugated back to MPA and reabsorbed in the colon through the action of glucuronidase shed by gastrointestinal tract bacteria (Bullingham et al., 1996b; Naderer et al., 2005). Biliary excretion of MPA/MPAG and subsequent distal absorption and reabsorption are likely to require several transport mechanisms including organic anion transporting polypeptides, MRP-2 and UGTs (Kuypers et al., 2005). EHR was observed in a small part of patients, but not significantly in the general population in our study, which was comparable to the previous studies (Armstrong et al., 2005; Wada et al., 2007; Cai et al., 2018; Zhang et al., 2018). The sparse blood sampling of 6–12 h may be related to the difficulty of noticing EHR in our patient population. Another possible reason was that the influence of intestinal bacteria arising from the uses of antibiotics in the early stage, which leads to the decrease of MPAG in intestinal de-glucuronic acid and the corresponding decrease of MPA re-entering the circulation and contributes to the relatively lower MPA-AUC_0–12h_ in our population as well (Naderer et al., 2005; Taylor et al., 2019). Consistently with the results of healthy Chinese population, no difference in PK parameters was observed between dispersible tablets and capsules in our study, which indicates the interchangeability of two formulations in some cases (Zhang et al., 2010). The parameters of T_max_ and MRT_0–12h_ in our MMFdt patients were similar to Japanese heart transplant cohort treated with MMFc (Wada et al., 2007). However, C_max_ and AUC_0–12h_ were different with Wada and Kaczmarek’s study in MMFc combined with TAC (Wada et al., 2007; Kaczmarek et al., 2008). The discrepancies may correlate with dosages, pathophysiological factor and concomitant medication during the period of PK investigation.

Given that MPA-AUC_0–12h_ is related to clinical outcomes, LSS for MPA-AUC_0–12h_ estimation is used to predict exposure for convenient use. MRA and the maximum a posteriori Bayesian method are two common methods used for establishing LSS model, while MRA is easier to operate with a basic statistical program. Since LSS derived from MRA are not dependent on the PK model of the drug and extensive knowledge about PKs of the medication is not required for use, LSS method can be easily incorporated into routine clinical practice. However, adherence to exactly sampling times is important to LSS because MRA depends on timed concentrations to predict AUC. Deviation of sampling time may adversely affect accuracy of the LSS model. Adherence to a strictly sampling time is usually difficult in clinical settings and thus limits the utility of MRA (van Warmerdam et al., 1994; Loh et al., 2007). Moreover, MRA is only applicable for the same dosage regimen in target population and has to be validated with a separate group (van Warmerdam et al., 1994; Loh et al., 2007). Conversely, Bayesian analysis doesn’t need to collect samples at specific times. Also, predictive performance of the LSS can be improved by continually updating data as more patient-specific data become available. Furthermore, patient demographic and clinical characteristics, in addition to pharmacokinetic data, can also be included to enhance predictive capability of the model. Besides prediction of AUC, other PK parameters can also be derived simultaneously, adding to efficiency and utility of analysis (Ting et al., 2006; Loh et al., 2007; Bruchet and Ensom, 2009). However, Bayesian analysis requires specific software and the process is complicated. The accuracy of its PK parameters of the specific medication is required for initial prediction and may not be readily available. It may cause large errors in estimating the overall parameters for a small test group (Loh et al., 2007). In the present study, we used MRA to develop an MPA-AUC_0–12h_ prediction model, and the 4-point model using 0.5, 2, 4, 6 h can accurately estimate of MPA-AUC_0–12h_ based on the result of *R* ^2^, MPE, MAPE and the difference between LSS and full AUC values. LSS model was better in predicting MPA-AUC_0–12h_ than a single-point time concentration, which was consistent with the previous results of Wada et al. and Kaczmarek et al. (Wada et al., 2007; Kaczmarek et al., 2008). Despite the reduced number of sampling in the 4-point model, it still requires a long sampling time of 6 h. Since patients are hospitalized during the early period of heart transplantation, the developed model is available for MPA TDM in such populations.

The common immunosuppressive regimen after heart transplantation is MMFdt or MMFc in combination with CNIs and prednisone. Considering the adverse reactions of CsA, TAC is now more widely used. Previous studies have demonstrated that MPA-AUC could be significantly affected by CsA due to HER (Armstrong et al., 2005; Wada et al., 2007; Zhang et al., 2018). CsA inhibits biliary secretion of MPAG by MRP-2 transporter (van Gelder et al., 2001; Kobayashi et al., 2004), leading to impaired excretion of MPAG in the bile and reducing enterohepatic recirculation of MPAG back to MPA (van Gelder et al., 2001; Hesselink et al., 2005). Therefore, the prediction model of MPA combined with CsA is not applicable for patients with combination of MPA and TAC. Limited models were reported when MMFc was combined with TAC in heart transplant recipients. Wada et al. developed a 3-time-point model using C_1_, C_2_ and C_4_ for predicting the full MPA-AUC_0–12h_ but it’s not reliable on clinic (Wada et al., 2007). (Kaczmarek et al., 20018) developed three estimation models by a case resampling bootstrap method and a 3-(C_0.5_, C_1_ and C_2_) and a 2-(C_0.5_ and C_2_) time-point model were more applicable to clinical practice (Kaczmarek et al., 2008). Woillard et al. developed 2 models by Bayesian estimation in heart transplant recipients receiving MMF in combination with corticosteroids and either CsA, CsA + everolimus or TAC (Woillard et al., 2015). However, the model of MPA-AUC_0–12h_ estimation in heart transplant recipients receiving MMFdt in combination with TAC has not yet been reported. In the study, we firstly developed and validated a 4-point time model to estimate MPA-AUC_0–12h_ in heart transplant patients with MMFdt combined with TAC.

As an exploratory study, we investigated the suitability of these published models to extrapolate into our MMFc patients. Six patients receiving MMFc was used to externally validate the models reported by (Kaczmarek et al., 2008). The MPE and MAPE values and consistency of these three models were not satisfied (Supplementary Figures S2, S3; Supplementary Table S1), indicating that these three models were not suitable for our patients receiving MMFc. Small data may be a limitation of the external validation and a study with large sample size is necessary for further study. The data of six MMFc patients was also used to validate the final LSS model in heart transplantation. The results presented that the established 4-point model can be extrapolated to MMFc patients when the PK parameters of MMFc and MMFdt are not notably varied. However, large population is needed to validate our findings that LSS models can be used in both MMFc and MMFdt.

Although the results of the model validation in our research were acceptable, the metrics used for evaluation of the LSS should be extended to more clinically relevant metrics. If a 20% difference is identified as the clinical acceptance of between full curve and LSS MPA-AUC_0–12h_, a considerable difference was observed between the full curve estimate and the LSS estimate for some patients (Supplementary Figures S4–S6). In the LSS models, the number of patients fell outside the 20% MPA-AUC_0–12h_ difference between full curve and LSS were 56.67% (17/30) in model 1, 33.33% (10/30) in model 2, 30.00% (9/30) in model 3, 16.67% (5/30) in model 4, 3.33% (1/30) in model 5, respectively. This finding indicated that the more point enrolled, the better predictive ability of the models was. AUC estimation using a 4-point LSS model is likely to lead to inaccurate estimation and outliers. However, such outliers really exist in clinic. Therefore, clinicians should first consider whether the patients belong to the high-risk group and the LSS model is suitable. If a 4-point LSS model is not applicable, increased sample points is recommended, such as using a 5-point LSS prediction model or adopting full-point sampling for estimation.

The present study had some limitations. Firstly, MPA samples were collected from a single center and a relatively small number of heart transplant patients after a short-term administration of MMFdt or MMFc. Multi-center studies with large scales should be warranted in the future. Secondly, blood samples were restricted to the first two post-transplant weeks and the MPA concentration may be influence by pathological characteristics of the patient in early post-transplant period. The LSS equation may not extrapolate in long-term follow-up after heart transplantation. Thirdly, no clinical outcomes were reported in the study. It is unknown whether the target range of 30–60 μg·h/mL for the MPA-AUC_0–12h_ is feasible for our population. Further studies should be conducted to investigate the relationship between the MPA-AUC_0–12h_ and the risk of rejection and adverse reactions in Chinese heart transplant recipients.

## Conclusion

In conclusion, a large inter-individual variability was observed in PK characteristics of MPA after multiple doses of MMFdt in Chinese adult heart transplant recipients. No significant difference was observed in the PK profiles of MMFdt and MMFc in our population. We developed a predictive equation for the estimation of full MPA-AUC_0–12h_ in the Chinese heart transplant recipients receiving MMFdt and TAC and validated the model in MMFdt and MMFc patients. 4-point LSS equation was chosen as the best predictive equation for estimation, and MPA-AUC_0–12h_ = 8.424 + 0.781 × C_0.5_ + 1.263 × C_2_ + 1.660 × C_4_ + 3.022 × C_6_ (*R* ^2^ = 0.844). The newly established LSS equation with a good consistency is able to be applied in Chinese adult heart transplant recipients receiving MMFdt or MMFc and TAC. Attentions should be paid when using the equation due to the limitation of lacking proper validation in both internal and external cohorts.

## Data Availability

The raw data supporting the conclusion of this article will be made available by the authors, without undue reservation.
