# Supplementary material for: Limited Sampling Strategy for Estimation of Mycophenolic Acid Exposure in Adult Chinese Heart Transplant Recipients
Source: Front Pharmacol. 2021 Apr 12;12:652333. doi: 10.3389/fphar.2021.652333 (PMC8072337; doi:10.3389/fphar.2021.652333)
Supplement: Supplementary file 1 [file datasheet1.docx]

**
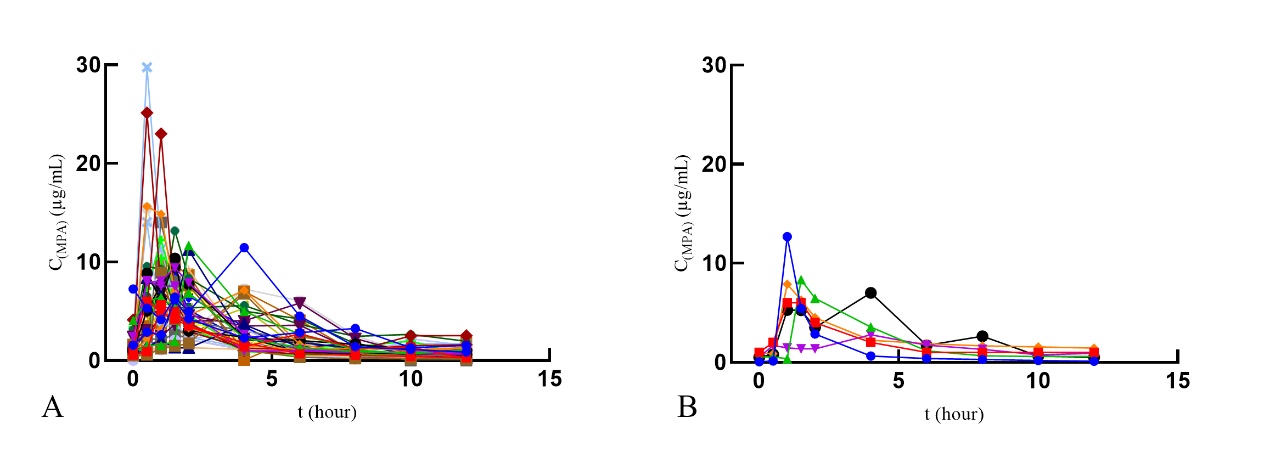
**

**Supplementary Figure S1.** Plasma concentration–time curves of MMFdt patients (n=42) (A), MMFc group (n=6) (B) in heart transplant recipients. MMFdt, mycophenolic mofetil dispersible tablets; MMFc, mycophenolate mofetil capsule; MPA, mycophenolic acid; C_(MPA)_, concentration of MPA; t, time.

**
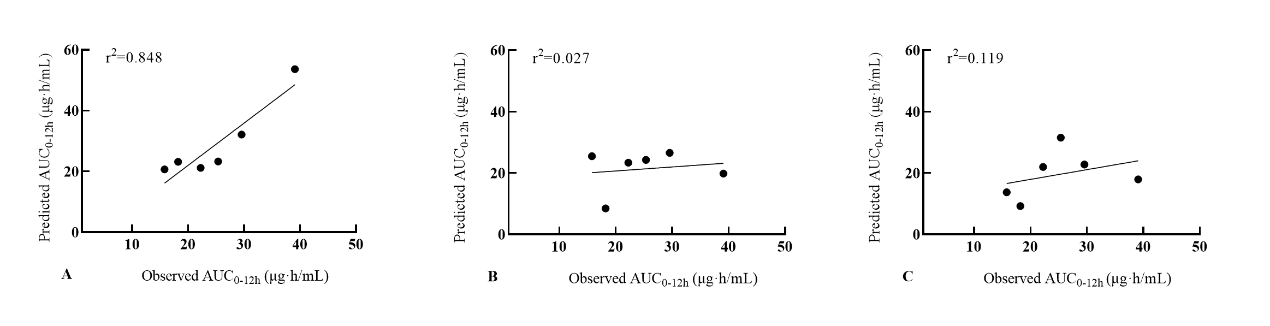
**

**Supplementary Figure S2.** Passing–Bablok regression analysis between the observed AUC_0-12h_ and the predicted AUC_0-12h_ of MPA in model a (A), model b (B), model c (C) using MMFc group. model a: AUC =1.25×C_1_ + 5.29×C_4_ + 2.90×C_8_ + 3.61×C_10_ (R^2^ = 0.95); model b: AUC = 1.09×C_0.5_ + 1.19×C_1_ + 3.60×C_2_ (R^2^ = 0.84); model c: AUC = 1.65×C_0.5_+4.74×C_2_(R^2^=0.75); AUC_0-12h_, the area under the 12-hour concentration-time curve; MPA, mycophenolic acid; MMFc, mycophenolate mofetil capsule.


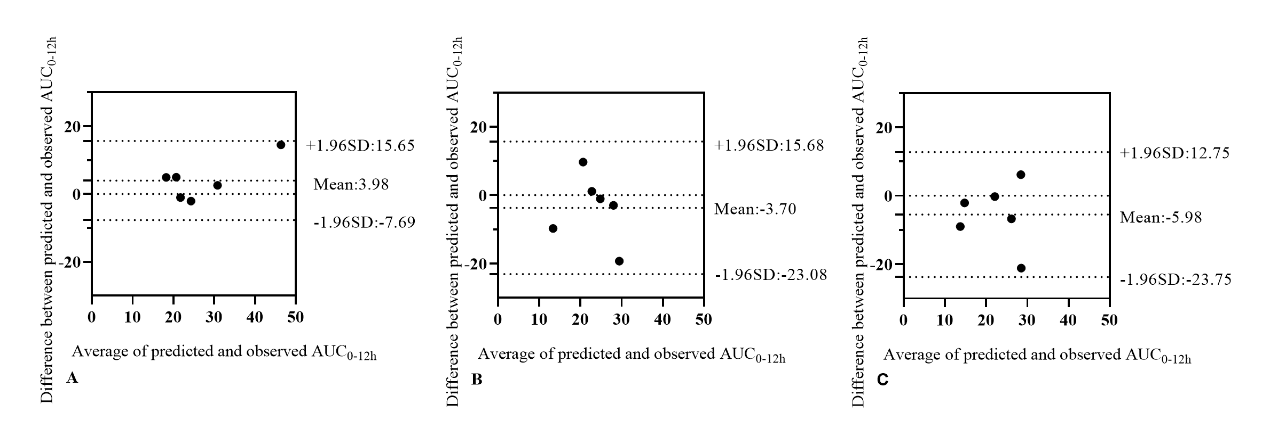


**Supplementary Figure S3.** Bland–Altman plot of differences between the observed AUC_0-12h_ and the predicted AUC_0-12h_ of MPA in model a (A), model b (B), model c (C) using MMFc group. model a: AUC =1.25×C_1_ + 5.29×C_4_ + 2.90×C_8_ + 3.61×C_10_ (R^2^ = 0.95); model b: AUC = 1.09×C_0.5_ + 1.19×C_1_ + 3.60×C_2_ (R^2^ = 0.84); model c: AUC = 1.65×C_0.5_+4.74×C_2_(R^2^=0.75); AUC_0-12h_, the area under the 12-hour concentration-time curve; MPA, mycophenolic acid; MMFc, mycophenolate mofetil capsule.


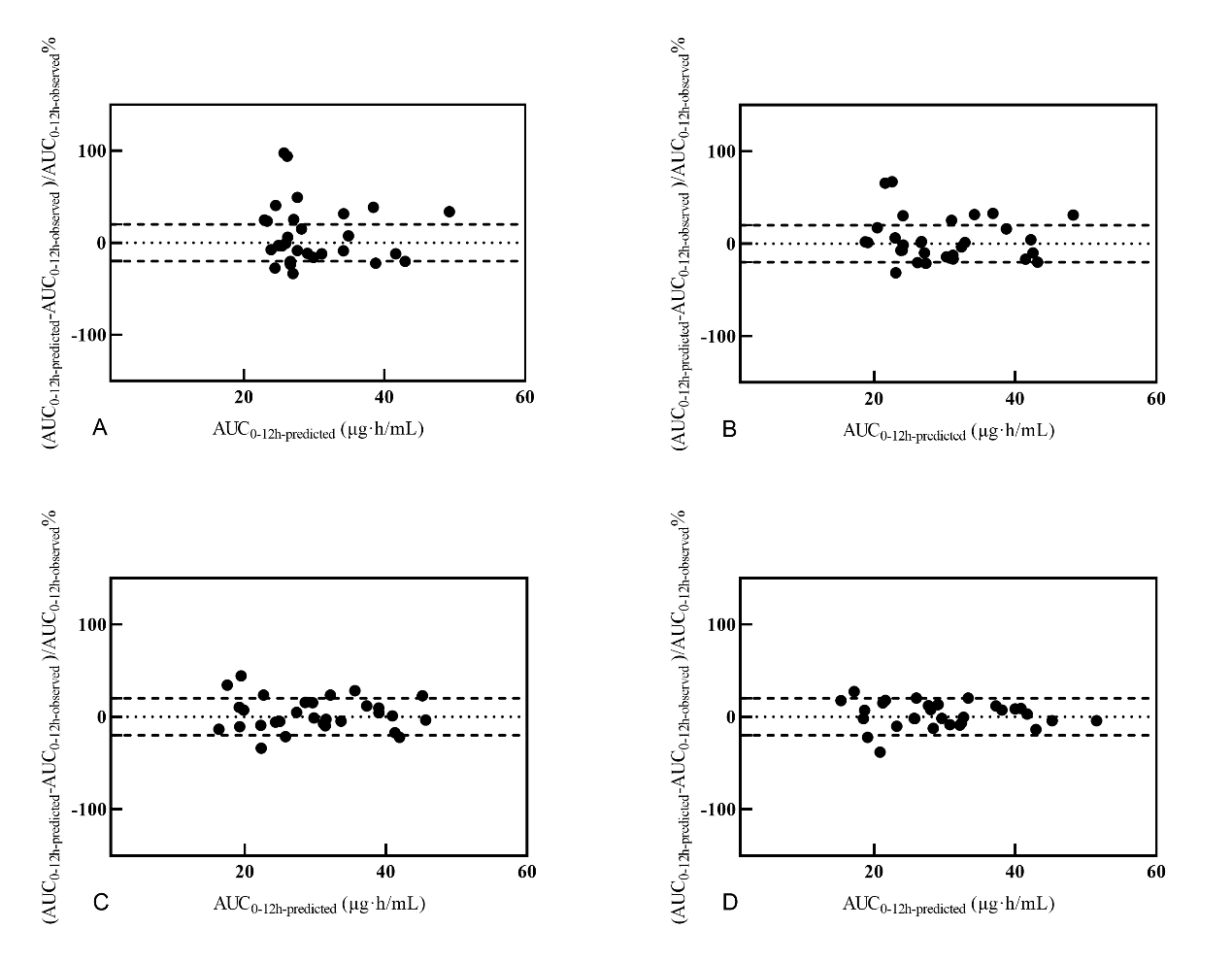


**Supplementary Figure S4.** Clinical acceptance (percentage of patients are at risk of receiving an incorrect dosing advice) of model development group. Including model 1 (A), model 2(B), model 3(C) and model 4 (D). The dotted line represents 20% MPA-AUC_0-12h_ difference between full curve and LSS, which is the clinical acceptance of the model. AUC_0-12h_, the area under the 12-hour concentration-time curve; MPA, mycophenolic acid; MPA-AUC_0-12h_-observed: the value of measured MPA-AUC_0–12h_; MPA-AUC_0-12h_-predicted: the value of predicted MPA-AUC_0–12h_.

**
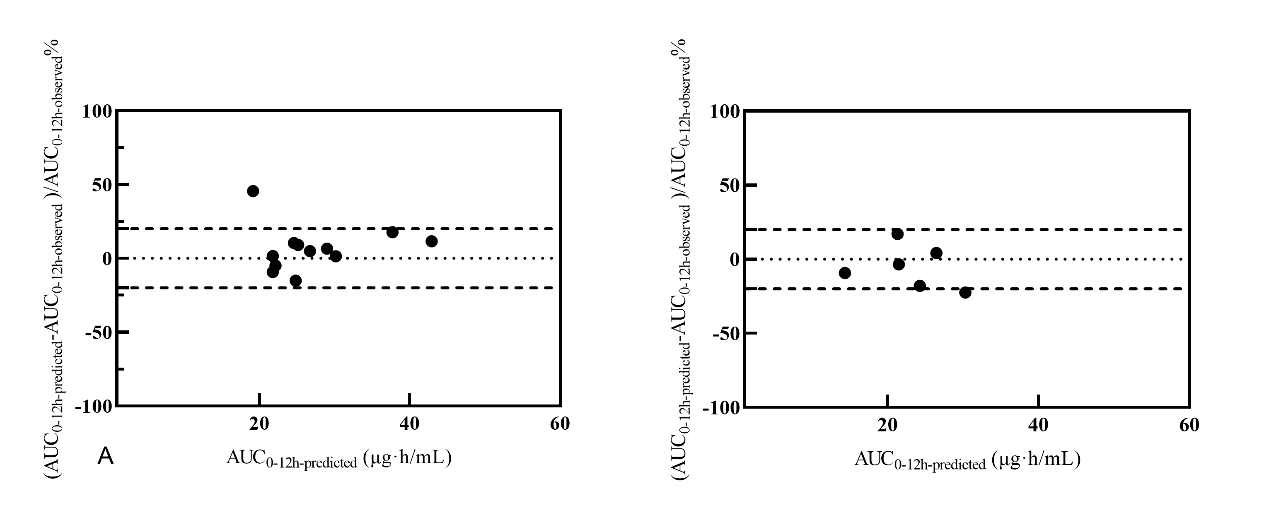
**

**Supplementary Figure S5.** Clinical acceptance (percentage of patients are at risk of receiving an incorrect dosing advice) of model validation group (A) and MMFc group (B) by model 4. The dotted line represents 20% MPA-AUC_0-12h_ difference between full curve and LSS, which is the clinical acceptance of the model. AUC_0-12h_, the area under the 12-hour concentration-time curve; MPA, mycophenolic acid; MMFc, mycophenolate mofetil capsule; MPA-AUC_0-12h_-observed: the value of measured MPA-AUC_0–12h_; MPA-AUC_0-12h_-predicted: the value of predicted MPA-AUC_0–12h_.

**
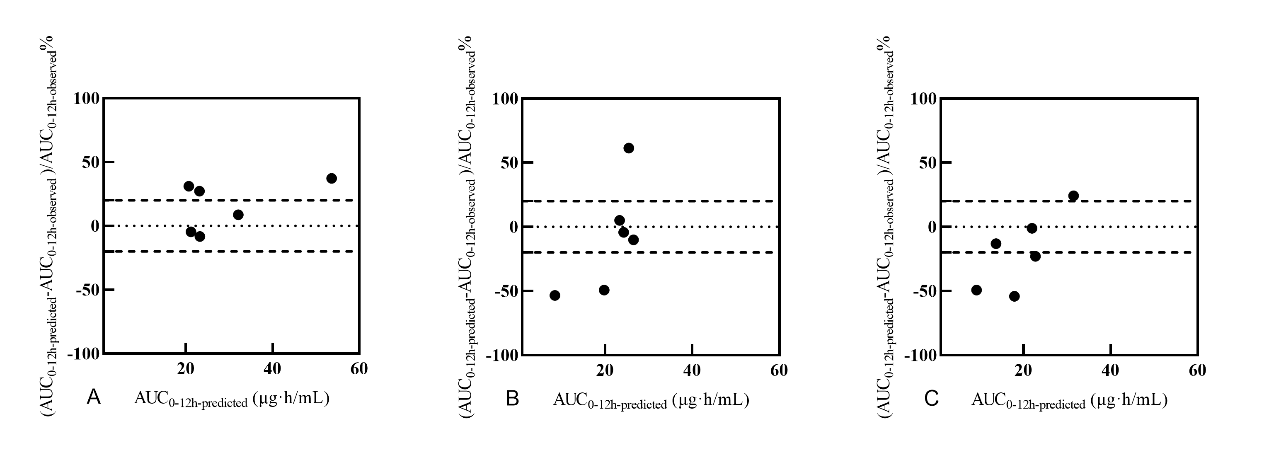
**

**Supplementary Figure S6.** Clinical acceptance (percentage of patients are at risk of receiving an incorrect dosing advice) of model a (A), model b (B), model c (C) using MMFc group. model a: AUC =1.25×C_1_ + 5.29×C_4_ + 2.90×C_8_ + 3.61×C_10_ (R^2^ = 0.95); model b: AUC = 1.09×C_0.5_ + 1.19×C_1_ + 3.60×C_2_ (R^2^ = 0.84); model c: AUC = 1.65×C_0.5_+4.74×C_2_(R^2^=0.75). The dotted line represents 20% MPA-AUC_0-12h_ difference between full curve and LSS, which is the clinical acceptance of the model. AUC_0-12h_, the area under the 12-hour concentration-time curve; MPA, mycophenolic acid; MMFc, mycophenolate mofetil capsule; MPA-AUC_0-12h_-observed: the value of measured MPA-AUC_0–12h_; MPA-AUC_0-12h_-predicted: the value of predicted MPA-AUC_0–12h_.
