# Supplementary material for: Limited Sampling Strategy for Estimation of Mycophenolic Acid Exposure in Adult Chinese Heart Transplant Recipients
Source: Front Pharmacol. 2021 Apr 12;12:652333. doi: 10.3389/fphar.2021.652333 (PMC8072337; doi:10.3389/fphar.2021.652333)
Supplement: Supplementary file 2 [file table1.docx]

**Supplementary Table S1.** External validation of model a, model b, model c using MMFc group

| Parameter | r^2^ | MPE (%) | MAPE (%) | Difference between LSS and full AUC values | | |
| --- | --- | --- | --- | --- | --- | --- |
|  |  |  |  | Within ±*15%* | Within ±*20%* | Within ±*25%* |
| model 4 (present study) | 0.800 | -5.25±14.62(-22.44, 17.15) | 12.41±7.88(3.37, 22.44) | 3 | 5 | 6 |
| model a (Kaczmarek, et al.,2008) | 0.848 | 15.25±17.64(-8.24, 37.23) | 19.54±12.73(4.63, 37.23) | 3 | 3 | 3 |
| model b (Kaczmarek, et al.,2008) | 0.027 | -8.41±38.24(-53.37, 61.44) | 30.58±24.46(4.21, 61.44) | 3 | 3 | 3 |
| model c (Kaczmarek, et al.,2008) | 0.119 | -19.34±27.05(-54.07, 24.31) | 27.44±18.77(1.13, 54.07) | 2 | 2 | 4 |

model a: AUC =1.25×C_1_ + 5.29×C_4_ + 2.90×C_8_ + 3.61×C_10_ (R^2^ = 0.95); model b: AUC = 1.09×C_0.5_ + 1.19×C_1_ + 3.60×C_2_ (R^2^ = 0.84); model c: AUC = 1.65×C_0.5_ + 4.74×C_2_ (R^2^ = 0.75); MMFc, mycophenolate mofetil capsule; r^2^, correlation coefficient by Passing-Bablok regression; MPE, mean prediction error; MAPE, mean absolute prediction error.

REFERENCES

Kaczmarek I., A. K. Bigdeli, M. Vogeser, T. Mueller, A. Beiras-Fernandez, P. Kaczmarek, et al.(2008).Defining algorithms for efficient therapeutic drug monitoring of mycophenolate mofetil in heart transplant recipients.*Ther Drug Monit*.30,419-427.doi:10.1097/FTD.0b013e31817d7064
